# Effects of diet and castration on fatty acid composition and volatile compounds in the meat of Korean native black goats

**DOI:** 10.5713/ab.22.0378

**Published:** 2023-01-11

**Authors:** Jinwook Lee, Hye-Jin Kim, Sung-Soo Lee, Kwan-Woo Kim, Dong-Kyo Kim, Sang-Hoon Lee, Eun-Do Lee, Bong-Hwan Choi, Farouq Heidar Barido, Aera Jang

**Affiliations:** 1 Animal Genetic Resources Research Center, National Institute of Animal Science, Hamyang 50000, Korea; 2 Department of Applied Animal Science, Kangwon National University, Chuncheon 24341, Korea; 3 Department of Agricultural Biotechnology, Center for Food and Bioconvergence, and Research Institute of Agriculture and Life Science, Seoul National University, Seoul 08826, Korea; 4 Department of Animal Science, Faculty of Agriculture, Universitas Sebelas Maret, Surakarta 57126, Indonesia

**Keywords:** Feeding Regimes, Goat, Intramuscular Fatty Acids, Meat Quality, Rumen Microbial Populations, Volatile Compounds

## Abstract

**Objective:**

This study determined the effects of dietary treatments and castration on meat quality, fatty acids (FAs) profiles, and volatile compounds in Korean native black goats (KNBG, *Capra hircus coreanae*), including the relationship between the population of rumen microbiomes and meat FA profiles.

**Methods:**

Twenty-four KNBG (48.6±1.4 kg) were randomly allocated to one of four treatments arranged into a 2×2 factorial structure. The factors were dietary forage to concentrate ratio (high forage [HF, 80:20] and low forage [LF, 20:80]), and a castration treatment (castration [CA] vs non-castration [NCA]).

**Results:**

Among meat quality traits, the CA group exhibited a higher percentage of crude fat and water holding capacity (p<0.05). The profiles of the saturated fatty acid (SFA) in meat sample derived from CA KNBG showed a significantly lower percentage compared to NCA individuals, due to the lower proportion of C14:0 and C18:0. Feeding a high-forage diet to KNBG increased the formation of C18:1n7, C18:3n3, C20:1n9, C22:4n6 in meat, and polyunsaturated fatty acid (PUFA) profiles (p<0.05). Consequently, the n6:n3 ratio declined (p<0.05). There was an interaction between dietary treatment and castration for formation of C20:5n3 (p<0.05), while C18:1n9, C22:6n3, monounsaturated fatty acid (MUFA) and the MUFA:SFA ratio were influenced by both diet and castration (p<0.05). Nine volatile compounds were identified and were strongly influenced by both dietary treatments, castration (p<0.05), and their interaction. In addition, principal component analysis (PCA) revealed distinctly different odor patterns in the NCA goats fed LF diets. Spearman correlation analysis showed a high correlation between rumen bacteria and meat PUFAs.

**Conclusion:**

These results suggest the essential effects of the rumen microbial population for the synthesis of meat FAs and volatile compounds in KNBG meat, where dietary intake and castration also contribute substantially.

## INTRODUCTION

Goat meat is an important protein source in many countries, due to fewer limitations to consumers from varied religious and cultural backgrounds. It is also appealing to health-conscious consumers owing to its low cholesterol and saturated fatty acid (SFA), and high proportion of polyunsaturated fatty acids (PUFAs) and mineral content, compared to beef or pork [1]. In addition, because goats adapt well to many environments, supply chain continuity and maintenance can be achieved more easily [2]. Nevertheless, the oxidized (fat-like) and lamby (sheep-like) oftentimes adhere as the main characteristics of goat meat, thus reduces its palatability [3]. However, modification of physicochemical properties, especially fatty acid (FA) composition can mitigate these negative attributes. The alteration of intramuscular FAs, which are strongly associated with volatile compound profiles are thought to improve the olfactory perceptions of consumers [4]. Madruga et al [5] elucidated that the alkanal and alkenals of the aldehydes that mainly responsible for the green, rancid, metallic, and oxidized flavor are generated from the oxidation of the C18 PUFAs, especially that of linoleic and linolenic acid, and C20:4n6 (arachidonic acid). Thus, extensive efforts to improve meat flavor have continuously performed through the modification of meat FAs.

Intramuscular FA composition is influenced by various factors, including genotype, age, sex, and diet [5–7]. Particularly in ruminant animals, its composition is also strongly determined by the interaction between diet and the rumen microbiome [8]. Incorporated feeds that enter the rumen experience biodegradation and fermentation, and are consequently converted into metabolic end products, including volatile fatty acids (VFAs) by rumen microbes [9]. These rumen microbes synthesize a variety of FAs, including those do not present in feed, using VFAs, which are then transferred to the lower gut to provide precursors for *de novo* FA synthesis for muscle tissue [10]. Earlier studies have reported that biohydrogenating bacteria, such as *Butyrivibrio* spp. and *Propionibacterium* spp. convert dietary PUFAs to various biohydrogenation intermediates, including conjugated linoleic acid and SFA [11,12].

Castration in ruminant animals is another critical contributing factor for the improvement of both animal performance and meat quality. The state of hormonal changes in castrated animals have been reported to modify metabolic status in livestock, and thus promote the efficiency of feed conversion [13]. Consequently, meat derived from non-castrated rams differs in tenderness and unsaturated fatty acid (UFA) proportions compared to that of females and [14]. In addition, meat of castrated goats has been reported to contain higher proportions of UFA and PUFA, along with lower branched chain fatty acids (BCFA), which are associated with its distinctive odor and off-flavor [11]. However, the correlations between rumen microbes and FA profiles, and how these are influenced by diet and castration, are still unclear.

The purpose of this study was to determine the effects of a dietary treatment with different forage to concentrate ratios, together with a castration treatment on meat quality, FA profiles, and volatile compounds in the meat of an economically important breed, the Korean native black goat (KNBG; *Capra hircus coreanae*), which is the predominant indigenous goats population in Korea, as well as to investigate the correlations between rumen microbial abundance and FA profiles of meat using high-throughput sequencing techniques.

## MATERIALS AND METHODS

### Study animals, diet treatments and sampling

Twenty-four mature KNBG (body weight, 48.6±1.4 kg; age, 4.8±1.2 years) were used in a three-month (12 week) feeding trial. All animals were randomly allocated to one of four treatments arranged in a 2×2 factorial structure. The factors were dietary forage to concentrate ratio (high forage [HF, 80:20] and low forage [LF, 20:80]) and castration (castration [CA] vs non-castration [NCA]) with chopped alfalfa hay as the sole forage. Animals were housed in individual pens (1.2 m ×0.9 m) and fed twice a day at 8 AM and 4 PM. Animals were each given free access to feed and water throughout the experimental period. The animal care and use protocols were followed under approval of the Institutional Animal Care and Use Committee of the NIAS, RDA, Republic of Korea (NIAS-2019-1545).

After the feeding trial, the animals were weighed and stunned using an electrical stunner (approximately 210 V). After stunning, goats were slaughtered at the slaughtering house of the National Institute of Animal Science (NIAS) in Rural Development Administration (RDA) according to the standard procedures. Goat rumen samples were collected and stored at −80°C for microbial analysis. Carcasses were weighed and stored in a cold room (4°C) for 24 h prior to carcass dissection. After boning, excised goat loin (GL) samples were vacuum-packed and stored at −20°C for further analysis.

### Physicochemical properties

The proximate composition of GL samples was determined using the Association of Official Agricultural Chemists (AOAC) method [15] with some modifications. Moisture content was analyzed by oven drying at 105°C to a constant weight, and crude protein content was analyzed using the Kjeldahl method. The crude fat percentage was analyzed by using Soxhlet extraction method with diethyl ether as the solvent, where the crude ash was analyzed in a furnace at 550°C for 12 h. Color measurements of the KNBG loin were performed in triplicate using a Minolta chromameter (Model CR-300; Minolta Co., Osaka, Japan), and the Commission Internationale de l’Eclairage (CIE) color values for lightness (CIE L*), redness (CIE a*), and yellowness (CIE b*) were measured. The chromameter was standardized using a white calibration plate (Y = 93.6, x = 0.3134, y = 0.3194).

For pH, 10 g of each sample was blended with 90 mL distilled water for 60 s in a homogenizer (PolyTron PT-2500 E; Kinematica, Lucerne, Switzerland). Subsequently the pH value of homogenates was determined using a pH meter (Orion 230A; Thermo Fisher Scientific, Waltham, MA, USA). For the water holding capacity (WHC) analysis, minced meat (0.5 g) was heated for 20 min at 80°C in a water bath and cooled to 23°C±2°C. After cooling, the samples were centrifuged at 2,000×g for 20 min and the total moisture was measured. The WHC values were calculated using the following equation: WHC (%) = [(total water content – separated water content)/total water content] × 100.

To determine the cooking loss, samples in vacuum-sealed bags were weighed and heated for 45 min until the temperature at the center of the meat reached 75°C±3°C. Cooking loss was calculated by converting the differences in weight before and after cooking into a percentage. To measure the shear force value, GL samples were placed in a polyethylene bag and heated in a water bath at 75°C to an internal temperature of 75°C±3°C. The loin samples were cut into 1×2×2 cm pieces and assessed using a texture analyzer (TA 1; Lloyd Instruments, Berwyn, PA, USA) with a V-shaped blade. The measurement conditions were a test speed of 50 mm/min and a 500 N load cell.

To measure the total bacterial counts, 10 g of loin sample was homogenized with 90 mL of peptone water in a stomacher bag (Bag Mixer 400; Interscience, St. Nom, France). After serial dilution, 1 mL of the diluent was loaded onto Petrifilms (3M Microbiology, St. Paul, MN, USA) for aerobic plate counts, which were incubated at appropriate temperatures according to the manufacturer’s instructions.

Volatile basic nitrogen (VBN) was measured in each KNBG sample, where 5 g of the sample was mixed with 50 mL of distilled water for 30 min and then filtered using filter paper (Whatman No. 1). The sample filtrate and 0.01 N H_2_SO_4_ were loaded onto a Conway unit (Sibata scientific technology, Co. Ltd., Tokyo, Japan) and incubated for 1 h at 25°C. After incubation, 10 μL of Brunswik indicator was added to the inner chamber of the Conway unit and titrated with 0.01 N NaOH. The VBN values were calculated using the following equation: VBN (mg/100 g) = 0.14×(*b*–*a*)×F/W×100×*d*, where a is the volume of 0.01 N NaOH added to the sample (mL), b is the volume of 0.01 N NaOH added to the blank (mL), F is the standard factor for 0.01 N NaOH, W is the sample weight (g), and *d* is the dilution factor.

### Fatty acid composition

Fatty acid analysis was performed on lipids extracted with Folch’s solution (2:1 mixture of chloroform and methanol, v/v) according to standard methods [16]. The lipid sample was placed in in a test tube, mixed with 1.5 mL of 0.5 N NaOH methanol solution, and heated at 100°C for 5 min. The samples were then mixed with 2 mL of 10% boron trifluoride solution and heated at 100°C for 2 min. Following the addition of 2 mL of iso-octane and 1 mL of saturated NaCl solution, the lipid samples were centrifuged at 783×g for 3 min. The fatty acid methyl ester was then analyzed using a gas chromatograph (6890N; Agilent Technologies, Wilmington, DE, USA) equipped with a flame ionization detector and capillary column (Omegawax 250 capillary column 30 m×0.25 mm×0.25 μm; Supelco, Bellefonte, PA, USA). Helium was used as the carrier gas at a flow rate of 1.2 mL/min. The temperatures of the oven and detector were 250°C and 260°C, respectively.

### Volatile compounds

Volatile compounds were analyzed using a HERACLES II electronic nose system (Alpha MOS, Toulouse, France) equipped with two flame ionization detectors and two capillary columns (MXT-5 and MXT-1701) in parallel. Goat meat samples were weighed (2 g) and immediately placed into 10 mL vials sealed with a silicon/Teflon septum and open-top caps. Subsequently, headspace samples were collected at 40°C for 5 min, and 5,000 μL of gas samples were injected for gas chromatography. The temperature of the trap was 40°C, and the column was held at 40°C for 5 s and raised to 270°C at 1.5°C/s, then to 270°C for 15 s. The volatile compounds were then identified using AlphaSoft software (AlphaSoft; Alpha MOS, France) with the AroChembase database.

### Statistical analysis

Data were analyzed using two-way analysis of variance in XLSTAT v. 2020.2.2 (Addinsoft, New York, NY, USA). A significance threshold of p<0.05 was used. Correlations between meat fatty acids and ruminal bacterial genera were obtained using Spearman’s correlation analysis. The resulting correlation matrix was visualized in heatmap format using the ‘psych’ package in R v. 4.0.2. To identify the difference in volatile compounds by diet and gender, multivariate statistical analysis was performed by principal component analysis. Analyses were performed using log-transformed and auto-scaled data using Metaboanalyst 5.0 (https://www.metaboanalyst.ca/).

## RESULTS AND DISCUSSION

### Physicochemical properties

The moisture and crude fat percentage were significantly affected by castration, where moisture content was markedly lower in meat of castrated goats compared to those without castration, regardless of dietary treatment (p<0.05; Table 1). In contrast, meat derived from castrated KNBG had a significantly higher fat percentage at any dietary treatment compared to that of non-castrated ones (p<0.05). Dietary treatment only significantly affected the crude ash percentage of the meat, where the high forage treatment had a higher score compared to the low forage treatment (p<0.05). In addition, these results did not reveal any interaction between castration and dietary treatments influencing the proximate composition of KNBG meat (p>0.05). Similarly, a meta-analysis by Sales et al [17] confirmed that the effect of castration to increases meat fat percentage. This phenomenon is thought to be caused by the reduction of testosterone, which affects fat metabolism, and the increase in the feed conversion ratio in castrated animals [18]. The results report here on proximate composition corroborate previous reports [19,20].

In terms of color measurements of KNBG meat, yellowness (CIE b*) was the only attribute to be influenced by castration. Meat samples derived from castrated KNBG were more intensely yellow in color compared to samples from non-castrated at low forage diet (p<0.05). Similarly, a higher yellowness value has been reported in castrated Boer crossbred wethers due to higher intramuscular fat content [21]. However, previous studies have also reported that the effects of castration on meat color were small [17] or insignificant [22] which is in agreement with our study, where the effect of both castration and dietary treatments on the lightness (CIE L*) and redness (CIE a*) of KNBG meat were not significant (p>0.05).

Our results show that the WHC of KNBG meat was the highest when castrated goats were fed with a high forage diet. WHC percentage was recorded at 51.04%, which was significantly higher than that of non-castrated individuals (46.55%) with the same diet treatment of high forage (Table 1). In contrast, this effect was not observed in the low forage treatment group (p>0.05). This finding was in line with previous work [23], where different muscle structures formed as a result of metabolic changes following castration were more influential than the forage to concentrate ratio. The WHC is an essential economic trait in meat, as the ability of muscle to bind and retain water during processing is related to sensory properties such as juiciness, texture, and flavor [24]. WHC is reported to be influenced by various factors, such as breed type, slaughter weight, and feeding regimes [18]. Further, in this experiment, the pH value ranged from 5.93 to 6.03, which was in line with previous results for goat meat [6,14]. However, neither castration nor dietary treatment (low vs high forage diet) significantly influenced the pH value of KNBG meat (p>0.05). Similarly, the cooking loss percentage, shear force value, aerobic plate counts, and the VBN concentration did not differ with dietary treatments or castration (p>0.05).

### Fatty acid composition

In animals with complex digestive systems, like ruminants, the formation of meat fatty acid is predominantly determined by two factors, namely, the rumen microbiome and fat deposition [25]. Therefore, it is possible to maintain the rumen microbial population through the dietary planning. In this study, oleic and palmitic acid were the main fatty acids in KNBG meat, occurring at approximately 28.63% to 38.56% and 23.02% to 24.73%, respectively. These findings are in agreement with previous studies on the longissimus muscle of goats [6] and lambs [18]. Dietary treatment significantly modified MUFA and PUFA percentages due to the changes in the individual FAs. The C18:1n9 percentage of the MUFA was the highest in castrated KNBG with a low forage diet and contributed around 38.56% to the total FA. In addition, these results revealed the considerable effect of castration, where, irrespective of dietary treatment, castrated KNBG showed significantly higher C18:1n9 percentage compared to that of non-castrated individuals. However, an interaction between dietary treatment and castration was not observed (p>0.05).

The higher proportion of C18:3n3 and C22:4n6 in the PUFA was observed in both a high forage and low forage diet, regardless of the castration. Further, these results showed a significant interaction between castration and dietary treatment on the formation of C22:6n3 FA, where castration tended to enrich the content of the C22:6n3 in the KNBG meat. Additionally, these results suggest that different dietary treatments may decide the FA composition of KNBG meat. Specifically, a high forage composition tended to increase the proportion of n-3 FAs. Furthermore, there were higher proportions of C18:1n7 under the high forage treatment, regardless of castration. Previous studies have found that C18:1n7 is an essential precursor for the conjugated isomers of linoleic acid (CLA) formation within rumen [26]. Approximately 20% to 30% of C18:1n7 absorbed by ruminants is converted into tissues by the enzyme delta-9 desaturase found in the rumen microbiome [27]. The treatment with higher concentrate, on the other hand, may increase the formation of n-6 PUFA precursors in cattle and sheep [28]. This phenomenon, however, was not clearly observed in the loin meat tested here, from either castrated or non-castrated KNBG (p>0.05).

Castrated KNBG potentially had differing metabolic processes. Regardless of dietary treatment, the total SFA in meat samples derived from castrated KNBG showed markedly lower percentage in comparison to that of non-castrated individuals (Table 2). The significantly lower proportions were mainly due to lower C14:0 and C18:0 (p<0.05). In contrast with SFA, MUFA showed significantly higher percentages compared to non-castrated samples (p<0.05), predominantly due to the increase of C16:1n7 and C18:1n9 FAs. In addition, although significant differences were observed in some of the individual PUFAs, such as C18:2n6, C18:3n6, C20:4n6, and C22:6n3, the total PUFA percentages were significantly lower only under low forage diet in meat samples derived from castrated KNBG compared to non-castrated ones. This result indicates that dietary treatment is more influential rather than castration. Nevertheless, Pratiwi et al [29] suggested that the higher proportion of the UFA in castrated boer was possibly due to the more efficient conversion of the SFA into MUFA via enzymes, such as MUFA cis-9 C18:1. The finding in this study on FAs properties indicate that meat derived from castrated KNBG may be healthier for consumers.

Our results showed that feeding KNBG with a forage-based diet, apart from castration, increased the PUFA/SFA ratio and decreased the n-6 to n-3 ratio linearly. Earlier studies have also mentioned that the formation of more n-3 concentration in muscle tissues following higher forage diet is the foremost reason for this observation, while feeding with concentrate-based diet resulted in higher n-6 formation [30]. Therefore, these findings suggest that forage or pasture-based diets could help to provide healthier meat, particularly with respect to the fat composition. Our results are compliant with the suggested PUFA/SFA ratio in meat safe for consumption, which should be above 0.4.

### Volatile compounds

The flavor profile of meat is developed from a complex series of interactions between volatile compounds [31], and is one of the most essential factors that dictate the level of satisfaction for consumers. Although studies investigating the effect of different forage to concentrate ratio on ruminant animals are widely available, clear conclusions related to these topics are still difficult to draw. A previous study found that the forage/pasture-based diet remarkably increased the species-specific flavor intensity [31], while others reported that grain-based diet significantly intensified fat, distinctive, and species-specific flavor [32,33]. In this study, nine individual volatile compounds with distinct sensorial perception were recorded, where dichloromethane was the predominant compounds at any sample groups (Table 3). Both dietary treatment and castration significantly influenced the development of volatile compounds in KNBG meat, where a strong interaction was also observed for individual compounds. In addition, treatment with low forage diet tended to exhibit notably higher levels of volatile compounds (e.g., methyl propanoate, 1-propanol, 2-methyl-, [E]-2-penten-1-ol, 2,4-octadiene, chlorobenzene, m-xylene, 1,2-diethylbenzene) compared to that of the high forage diet, except for dichloromethane, which showed higher concentration in high forage diet (p<0.05). Furthermore, meat samples derived from non-castrated KNBG fed with low forage diet were observed to have the highest concentrations of volatile compounds (p< 0.05).

**Figure 1:**
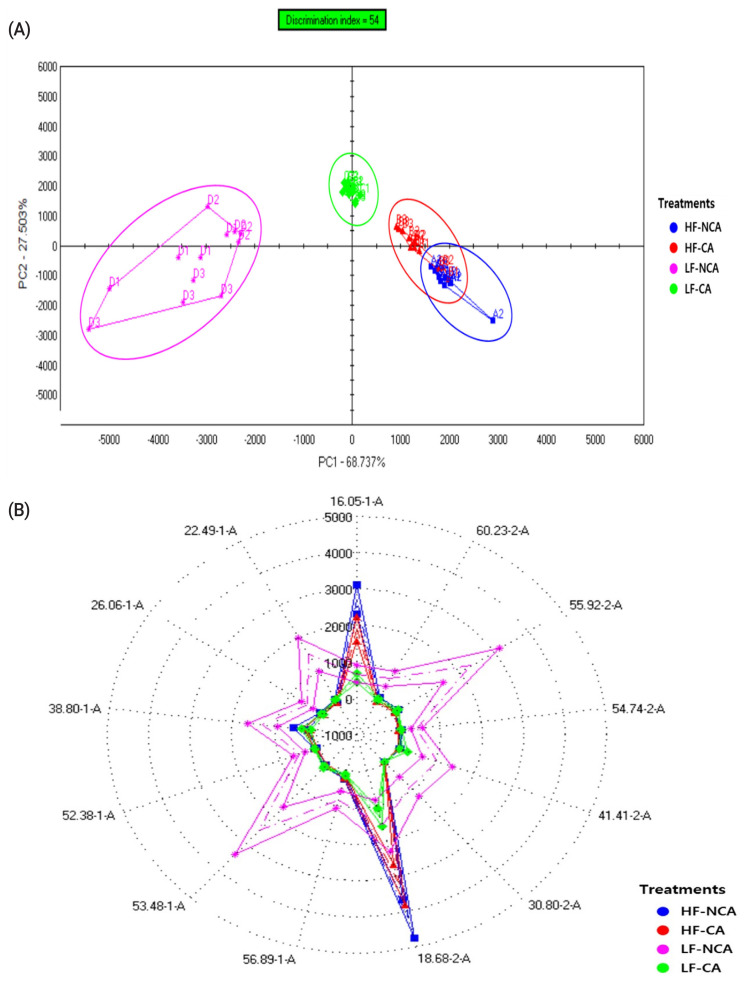
Principle component analysis plot (A) and radar plot (B) of volatile compounds of Korean native goat loin by diet and gender based on electronic nose signals.

The PCA indicated that the total variance explained was 68.737% for PC1 and 27.503% for PC2 (Figure 1A). These results suggest that feeding KNBG with low forage diet, regardless of the castration, might result in completely different odor perception, as meat derived from non-castrated animals solely clustered in the negative axis of the PC1, while the castrated ones mostly clustered in the positive axis of the PC2. Further, non-castrated KNBG fed with low forage diet exhibited the highest score for most of the sensors (Figure 1B), except for 16.05-1-A and 18.68-2-A sensors. For volatile compounds, dichloromethane and m-xylene were reported to be linked with the “strong lamb odor” and were influenced by dietary selection. Consistent with a previous report [33], feeding ruminant animals with high portion of forage in the diets is associated with “grassy” and “gamey” flavor, whereas grain-based diets led to the development of “ruminant fat” and “roasted” flavors [13]. Moreover, in this study, the intensity of a strong “goaty” flavor was remarkably enhanced in meat derived from non-castrated KNBG fed with the low forage diet. Aside from the influence of fatty acid composition, this may be caused by metabolic differences due to the hormonal discrepancies causing distinct fat and connective tissue proportions, which in turn affect the flavor properties of meat. Previous work has revealed that hydrocarbons and ketones are more abundant in castrated goat meat, while aliphatic aldehydes are higher in non-castrated goat meat, possibly owing to the activity of testosterone, androsterone, and skatole [34].

### Correlations between rumen microbiota and production variables

**Figure 2:**
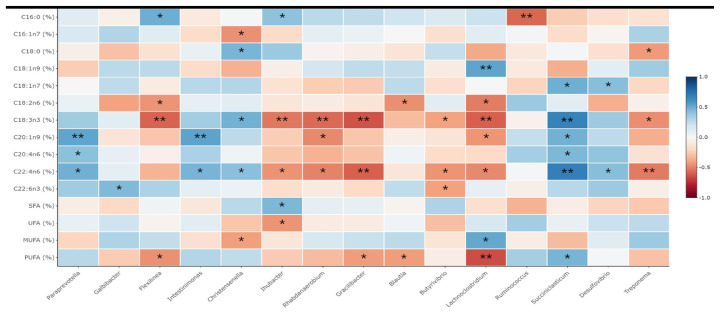
Correlation between rumen fermentation/intramuscular fatty acids profiles and genus abundance. Spearman non-parametric rank correlation matrix of the dominant bacterial genera across the rumen. The genera were included in the matrix if they were in at least 50% of goats and represented at least 0.1% of the bacterial community in at least one of the goats. The scale colors denote whether the correlation is positive (closer to 1, blue squares) or negative (closer to −1, red squares) between the bacteria and the efficiency parameters. Significance levels of correlations was expressed by asterisks (** for p<0.01, * for p<0.05).

The relationship between meat FA composition and rumen bacteria at the genus level was evaluated. There were significant interactions between the production of individual FAs and the rumen microbiome (Figure 2). Firstly, the proportion of the C16:0 positively correlated with the abundance of *Flexilinea* (Spearman’s ρ = 0.491; p = 0.015) and *Ihubacter* (Spearman’s ρ = 406; p = 0.049), while negatively correlated with the *Ruminococcus* (Spearman’s ρ = −0.575; p = 0.003). Secondly, C16:1n7 proportion was negatively correlated with the presence of the *Christensenella* (Spearman’s ρ = −0.455; p = 0.025), while the C18:1n9 formation positively correlated with *Lachonoclostridium* (Spearman’s ρ = −0.528; p = 0.008). In addition, the percentage of the C18:0 positively correlated with the abundance of *Christensenella* (Spearman’s ρ = 0.461; p = 0.023) and negatively correlated with that of *Treponema* (Spearman’s ρ = −0.429; p = 0.037). The formation of the C18:1n7 was positively correlated with that of *Succiniclasticum* (Spearman’s ρ = 0.487; p = 0.016) and *Desulfovibrio* (Spearman’s ρ = 0.426; p = 0.038), whereas C18:2n6 proportion was negatively correlated with the *Flexilinea* (Spearman’s ρ = −0.449; p = 0.028), *Blautia* (Spearman’s ρ = −0.463; p = 0.023), and *Lachnoclostridium* (Spearman’s ρ = −0.515; p = 0.035). Furthermore, the C18:3n3 proportion was positively correlated with the presence of the *Christensenella* (Spearman’s ρ = 0.477; p = 0.019) and *Succiniclasticum* (Spearman’s ρ = 0.673; p<0.001), and negatively correlated with that of *Flexilinea* (Spearman’s ρ = −0.589; p = 0.002), *Ihubacter* (Spearman’s ρ = −0.530; p = 0.008), *Rhabdanaerobium* (Spearman’s ρ = −0.570; p = 0.004), *Gracilibacter* (Spearman’s ρ = −0.633; p = 0.001), *Butyrivibrio* (Spearman’s ρ = −0.405; p = 0.049), and *Lachnoclostridium* (Spearman’s ρ = −0.602; p = 0.002). Further, the formation of the of C20:4n6 positively correlated with the abundance of *Paraprevotella* (Spearman’s ρ = 0.417; p = 0.043) and *Succiniclasticum* (Spearman’s ρ = 0.451; p = 0.027), while the C22:4n6 positively correlated with the *Paraprevotella* (Spearman’s ρ = 0.483; p = 0.017), *Intestinimonas* (Spearman’s ρ = 0.468; p = 0.021), *Christensenella* (Spearman’s ρ = 0.420; p = 0.041), *Succiniclasticum* (Spearman’s ρ = 0.685; p<0.001) and *Desulfovibrio* (Spearman’s ρ = −0.426; p = 0.038), and negatively correlated with the *Ihubacter* (Spearman’s ρ = −0.450 p = 0.027), *Rhabdanaerobium* (Spearman’s ρ = −0.499; p = 0.013), *Gracilibacter* (Spearman’s ρ = −0.595; p = 0.002), *Butyrivibrio* (Spearman’s ρ = −0.450; p = 0.028), *Lachnoclostridium* (Spearman’s ρ = −0.480; p = 0.018), and *Treponema* population (Spearman’s ρ = −0.521; p = 0.009). Moreover, the proportion of the C22:6n3 FAs positively correlated with the exsistence of the *Galbibacater* (Spearman’s ρ = −0.602; p = 0.002), while negatively correlated with that of *Butyrivibrio* (Spearman’s ρ = −0.602; p = 0.002) and *Oribacterium* (Spearman’s ρ = −0.602; p = 0.002). The result of this study found that the SFA percentages was positively correlated with the *Ihubacter* (Spearman’s ρ = 0.450; p = 0.028), while UFA had negative correlation with that of *Ihubacter* (Spearman’s ρ = −0.450; p = 0.028). MUFA positively correlated with the population of the *Lachnoclostridium* (Spearman’s ρ = 0.514; p = 0.010) and negatively correlated with the *Christensenella* (Spearman’s ρ = −0.414; p = 0.044). Finally, the PUFA percentages had positive correlation to that of *Succiniclasticum* (Spearman’s ρ = 0. 463; p = 0.023), while negatively correlated with the *Flexilinea* (Spearman’s ρ = −0.457; p = 0.025), *Gracilibacter* (Spearman’s ρ = −0.423; p = 0.039), *Blautia* (Spearman’s ρ = −0.423; p = 0.040), and *Lachnoclostridium* (Spearman’s ρ = −0.653; p = 0.001).

The relationship between meat FA composition and rumen bacteria at the genus level was clear, where ruminant products were influenced by both FA composition and biohydrogenating bacteria [13,25,35]. In our study, *Butyrivibrio* was negatively correlated with C18:3n3 and C20:4n6, which is consistent with previous study [25]. Rumen biohydrogenating bacteria represented by *Butyrivibrio* spp. detoxify dietary PUFA and convert UFA to SFA to provide FA synthesis precursors [12]. The metabolism of linoleic acid by *Butyrivibrio* results in the formation of trans-11-18:1 and cis-9, trans-11-18:2 as major intermediates. In our study, the population of *Paraprevotella*, *Intestinimonas*, *Christensenella*, and *Succiniclasticum* exhibited a positive correlation with that of PUFAs in meat derived from KNBG, which might be due to distinct differences in dietary PUFA intake. An earlier study found that ruminal bacteria, which are linked to propionate production and succinate metabolism, showed a strong relationship with biohydrogenation [14]. In this regard, *Paraprevotella*, succinate producing bacteria, and *Succinicalsticum*, which uses succinate to produce propionate, showed a positive correlation with trans 18:1 concentration in sheep due to higher intake of dietary PUFA. Further, butyrate-producing bacteria species, including *Intestinimonas* and *Christensenella*, were also correlated to the production of PUFAs in KNBG meat. This may be due to butyrate production and ruminal energy metabolism, which further influences adipose metabolism and CLA content in goats [36]. The abundance of *Christensenella* was also positively correlated with C18:0 content, which is consistent with previous study [37].

Notably, these results revealed a negative correlation of the *Treponema* population with C18:0, C18:3n3, and C20:4n6, which is consistent with previous results for sheep [37]. Early studies have reported that *Treponema* was negatively correlated with ruminal acetate and butyrate concentrations [38]. This species requires long-chain FAs for their growth and contributes to lipid metabolism [39]. *Lachnoclostridium* and *Blautia* also showed negative correlations with PUFAs, which might be related to ruminal VFAs concentrations. This genus mainly ferments polysaccharides to simple sugars that can be utilized as substrates for microbial growth and fermentation. Moreover, *Lachnoclostridium* could ferment lactate to VFAs, which could improve the ruminal papilla and mucosa. The genus *Gracilibacter* is the predominant bacteria found in lambs and musk deer. A previous study has reported that *Gracilibacter* was negatively correlated with ruminal butyrate concentration in cattle [37]. *Rhabdanaerobium* are rods with a gram-positive cell wall and obligate anaerobes. This genus of bacteria can utilize carbon and nitrogen sources as well as complex substrates, including peptone, starch, and yeast as energy sources [25]. However, the role of these bacteria in the rumen remains unclear. In addition, these results suggest that the presence of the *Butyrivibrio*, *Paraprevotella*, *Intestinimonas*, *Christensenella*, *Succiniclasticum*, *Gracilibacter*, *Rhabdanaerobium*, *Lachnoclostridium*, and *Treponema* might be correlated to the biohydrogenation and VFAs utilization in the rumen. Thus, production of meat FAs may be regulated through this phenomenon.

## CONCLUSION

The physicochemical properties, including fatty acid (FA) composition and volatile compounds, of meat derived from castrated or non-castrated Korean native black goat (KNBG) fed a diet of different forage levels were investigated. Castration significantly influenced meat quality attributes, including a higher percentage of fat and water holding capacity. The dietary treatments created distinct rumen microbial populations, resulting in differing production of individual FAs and volatile compounds. The n-3 FAs (C18:3n3, C22:6n3) were increased in castrated KNBG fed with a high forage diet, which implied healthier meat properties due to the linear decline of the n-6 to n-3 ratio. Moreover, the principal component analysis suggested there were distinct differences in odor intensity due to dietary treatments and castration. The results of this study provide important preliminary data for understanding the development of KNBG meat based on the correlation between rumen microbial population and the formation of meat FAs and volatile compounds. Further in-depth studies are necessary to investigate the specific mechanisms by which differing diets affect the rumen microbiome, focusing on biohydrogenation activity and its contribution to the synthesis of meat FAs in KNBG.

## Figures and Tables

**Table 1:** Effects of dietary treatment and castration on physicochemical properties of the Korean native black goats

Variables	High forage		Low forage		SEM	p-value		
		
	CA	NCA	CA	NCA		D	G	D×G
Proximate composition								
Moisture (%)	73.31^b^	76.25^a^	74.43^b^	76.36^a^	0.41	0.152	<0.001	0.237
Crude protein (%)	20.66	21.36	20.93	21.40	0.28	0.592	0.053	0.701
Crude fat (%)	5.44^a^	3.51^b^	5.72^a^	3.46^b^	0.18	0.525	<0.001	0.366
Crude ash (%)	1.08^a^	1.07^a^	1.01^b^	0.96^b^	0.02	0.001	0.297	0.464
Meat color								
CIE L	40.42	40.41	41.00	39.81	0.73	0.991	0.423	0.430
CIE a	24.30	24.51	25.46	23.95	0.47	0.529	0.185	0.081
CIE b	13.69^ab^	13.38^b^	14.38^a^	13.29^b^	0.30	0.327	0.030	0.213
Physicochemical characteristics								
pH	6.03	5.93	5.94	5.95	0.04	0.278	0.204	0.140
Water holding capacity (%)	51.04^a^	46.55^b^	47.55^b^	45.86^b^	1.18	0.092	0.017	0.249
Cooking loss (%)	32.66	34.66	32.27	34.65	1.31	0.880	0.109	0.888
Shear force (kgf)	6.79	7.21	6.71	6.92	0.16	0.249	0.059	0.520
APC (log CFU/g)	2.30	2.37	2.23	2.28	0.06	0.171	0.358	0.856
VBN (mg/100 g)	6.76	7.03	7.00	6.87	0.23	0.869	0.767	0.415

CA, castration; NCA, non-castration; SEM, standard error of the means; D, diet; G, gender; I, interaction; CIE, Commission Internationale de l’Eclairage; APC, aerobic plate counts; VBN, volatile basic nitrogen.

a,b Means within the same row with different letters are significantly different at p<0.05.

**Table 2:** Effects of dietary treatment and castration on fatty acid composition of the Korean native black goats

Variables (%)	High forage		Low forage		SEM	p-value		
		
	CA	NCA	CA	NCA		D	G	D×G
C14:0 (myristic acid)	1.78^b^	2.29^a^	1.75^b^	2.27^a^	0.13	0.831	0.001	0.988
C16:0 (palmitic acid)	23.02	24.23	24.00	24.73	0.63	0.254	0.141	0.712
C16:1n7 (palmitoleic acid)	1.44^ab^	1.18^b^	1.71^a^	1.28^b^	0.11	0.124	0.007	0.460
C18:0 (stearic acid)	15.72^b^	18.27^a^	14.44^b^	17.43^a^	0.55	0.066	<0.001	0.693
C18:1n9 (oleic acid)	32.68^b^	28.63^c^	38.56^a^	30.96^bc^	0.93	<0.001	<0.001	0.072
C18:1n7 (vaccenic acid)	2.27^a^	1.85^ab^	1.60^b^	1.45^b^	0.18	0.008	0.130	0.481
C18:2n6 (linoleic acid)	10.29^bc^	12.50^ab^	8.81^c^	13.26^a^	0.79	0.654	<0.001	0.170
C18:3n6 (γ-linoleic acid)	0.11^ab^	0.08^b^	0.16^a^	0.08^b^	0.02	0.233	0.015	0.265
C18:3n3 (α-linolenic acid)	1.83^a^	2.11^a^	0.59^b^	0.69^b^	0.14	<0.001	0.196	0.536
C20:1n9 (eicosenoic acid)	0.36^ab^	0.47^a^	0.28^b^	0.34^ab^	0.05	0.036	0.105	0.580
C20:4n6 (arachidonic acid)	7.21^a^	5.94^b^	6.08^b^	5.56^b^	0.37	0.056	0.026	0.316
C20:5n3 (eicosapentaenoic acid)	0.41^a^	0.25^b^	0.34^ab^	0.40^ab^	0.05	0.420	0.309	0.037
C22:4n6 (adrenic acid)	2.56^a^	2.08^a^	1.50^b^	1.44^b^	0.16	<0.001	0.125	0.216
C22:6n3 (docosahexaenoic acid)	0.32^a^	0.12^b^	0.18^b^	0.12^b^	0.03	0.033	<0.001	0.031
SFA	40.52^b^	44.79^a^	40.18^b^	44.43^a^	1.00	0.731	<0.001	0.988
UFA	59.48^a^	55.21^b^	59.80^a^	55.57^b^	1.00	0.738	<0.001	0.980
MUFA	36.76^b^	32.14^c^	42.16^a^	34.02^c^	0.91	0.001	<0.001	0.068
PUFA	22.73^a^	23.07^a^	17.64^b^	21.56^a^	1.29	0.019	0.115	0.183
MUFA/SFA	0.91^b^	0.72^c^	1.05^a^	0.77^c^	0.03	0.006	<0.001	0.107
PUFA/SFA	0.57	0.52	0.44	0.49	0.04	0.071	0.997	0.294
n6/n3 ratio	8.11^b^	8.42^b^	14.99^a^	16.94^a^	0.67	<0.001	0.109	0.239

CA, castration; NCA, non-castration; SEM, standard error of the means; D, diet; G, gender; I, interaction; SFA, saturated fatty acids; UFA, unsaturated fatty acids; MUFA, monounsaturated fatty acids; PUFA, polyunsaturated fatty acids; n6, fatty acid with the last double bond at 6th carbon from the methyl end; n3, fatty acid with the last double bond at 3rd carbon from the methyl end.

a–c Means within the same row with different letters are significantly different at p<0.05.

**Table 3:** Effects of dietary treatment and castration on volatile compounds of the Korean native black goats

Compounds	Sensory description	Column	RT (RI)	High forage diets		Low forage diets		SEM	p-value		
		
				CA	NCA	CA	NCA		D	G	D×G
Dichloromethane	ND	MXT-5	16.05 (505)	1,905.4^b^	2,714.2^a^	564.3^c^	665.7^c^	88.2	<0.001	<0.001	<0.001
		MXT-1701	18.68 (611)	3,234.6^b^	4,206.9^a^	1,331.7^c^	1,576.1^c^	165.6	<0.001	0.001	0.033
Methyl propanoate	Etheral, fruity, rum	MXT-5	22.49 (627)	26.3^b^	56.8^b^	91.2^b^	1,493.0^a^	78.6	<0.001	<0.001	<0.001
		MXT-1701	ND	ND	ND	ND	ND	-	-	-	-
1-Hydroxy-2-propanone	Caramelized, sweet	MXT-5	26.06 (664)	15.3^b^	43.3^b^	14.1^b^	437.7^a^	25.6	<0.001	<0.001	<0.001
		MXT-1701	ND	ND	ND	ND	ND	-	-	-	-
1-Propanol, 2-methyl-	Alcoholic, bitter, glue, leek	MXT-5	ND	ND	ND	ND	ND	-	-	-	-
		MXT-1701	30.80 (745)	0.0^b^	0.0^b^	0.0^b^	906.5^a^	53.6	<0.001	<0.001	<0.001
[E]-2-penten-1-ol	Grassy, green, mushroom	MXT-5	38.80 (769)	196.9^b^	375.8^b^	238.5^b^	1,274.1^a^	63.3	<0.001	<0.001	<0.001
		MXT-1701	ND	ND	ND	ND	ND	-	-	-	-
2,4-Octadiene	Glue, warm	MXT-5	ND	ND	ND	ND	ND	-	-	-	-
		MXT-1701	41.41 (825)	93.3^b^	108.3^b^	196.0^b^	1,070.8^a^	61.3	<0.001	<0.001	<0.001
Chlorobenzene	Etheral, floral, sweet	MXT-5	52.38 (866)	59.1^b^	53.4^b^	83.8^b^	481.3^a^	23.9	<0.001	<0.001	<0.001
		MXT-1701	54.74 (920)	18.7^b^	55.2^b^	70.3^b^	445.1^a^	22.7	<0.001	<0.001	<0.001
m-Xylene	Cold meat fat, plastic	MXT-5	53.48 (874)	128.2^b^	155.9^b^	187.8^b^	2,527.7^a^	132.1	<0.001	<0.001	<0.001
		MXT-1701	55.92 (928)	113.6^b^	171.4^b^	174.0^b^	2,342.4^a^	123.9	<0.001	<0.001	<0.001
1,2-diethylbenzene	Fatty, geranium, oily	MXT-5	56.89 (899)	175.7^b^	225.8^b^	140.3^b^	837.0^a^	38.4	<0.001	<0.001	<0.001
		MXT-1701	60.23 (959)	62.7^b^	129.1^b^	91.0^b^	745.1^a^	36.2	<0.001	<0.001	<0.001

RT, retention time (min); RI, retention index; SEM, standard error of the means; D, diet; G, gender; I, interaction; ND, not detected

a–c Means within the same row with different letters are significantly different at p <0.05
